# Multi-Drug Resistant Gram-Negative Sepsis in Neonates: The Special Role of Ceftazidime/Avibactam and Ceftolozane/Tazobactam

**DOI:** 10.3390/medicines12030017

**Published:** 2025-06-26

**Authors:** Niki Dermitzaki, Foteini Balomenou, Anastasios Serbis, Natalia Atzemoglou, Lida Giaprou, Maria Baltogianni, Vasileios Giapros

**Affiliations:** 1 Neonatal Intensive Care Unit, School of Medicine, University of Ioannina, 45500 Ioannina, Greece; n.dermitzaki@uoi.gr (N.D.); f.balomenou@uoi.gr (F.B.); md06684@uoi.gr (N.A.); md07243@uoi.gr (L.G.); mbalt@doctors.org.uk (M.B.); 2 Pediatric Department, School of Medicine, University of Ioannina, 45500 Ioannina, Greece; aserbis@uoi.gr

**Keywords:** neonatal sepsis, multidrug-resistant gram-negative bacteria, beta-lactams/beta-lactamase inhibitors, ceftazidime/avibactam, ceftolozane/tazobactam

## Abstract

Neonatal sepsis is a major cause of morbidity and mortality in neonates. A particular concern is the increasing prevalence of antibiotic-resistant strains among neonatal intensive care units (NICUs). Two novel beta-lactam/beta-lactamase inhibitors have recently been approved for use in neonates with multidrug-resistant infections: ceftazidime/avibactam and ceftolozane/tazobactam. These agents demonstrate efficacy against a range of multidrug-resistant gram-negative pathogens, including extended-spectrum beta-lactamases (ESBL)-producing and carbapenem-resistant *Enterobacterales*, as well as multidrug-resistant *Pseudomonas aeruginosa.* This narrative review aims to summarize the current knowledge concerning the utilization of ceftazidime/avibactam and ceftolozane/tazobactam in the NICU. According to the existing literature, both agents have been shown to be highly effective with a favorable safety profile in the neonatal population.

## 1. Introduction

### 1.1. Neonatal Sepsis

Sepsis is a major contributor to morbidity and mortality in the neonatal population. It is estimated that 1.3 million episodes of neonatal sepsis occur annually, resulting in more than 200,000 deaths [1,2]. Sepsis disproportionately affects premature and low-birth-weight neonates, who present the most vulnerable population due to the immaturity of their immune systems, the need for prolonged neonatal intensive care unit (NICU) stay, and the frequent need for invasive procedures and devices [3]. The prevalence of neonatal sepsis demonstrates considerable geographical variation, with low- and middle-income countries (LMICs) exhibiting higher incidence rates. A recent systematic review, predominantly comprising studies from LMICs, reported an incidence of 2824 cases of sepsis per 100,000 live births, accompanied by a mortality rate of 17.6% [4].

Neonatal sepsis is categorized as either early-onset sepsis (EOS) if it presents within the first 72 h of postnatal life or late-onset sepsis (LOS) if it presents after 72 h of postnatal life [5]. *Streptococcus agalactiae* and *Escherichia coli* are reported as the most common causative agents of EOS; however, a variety of pathogens may be involved, including *Staphylococcus aureus*, other *Streptococci*, *Enterococcus* spp., gram-negative *Enterobacterales*, *Listeria*, and fungi [3,6]. The pathogens involved in EOS are typically acquired vertically, either during labor from the maternal flora or in utero, hematogenously across the placenta, or ascending from the maternal genitourinary tract following rupture of membranes [7]. The pathogens involved in LOS are acquired horizontally from the NICU environment or the community. In the NICU, common sources of pathogens are contaminated surfaces and indwelling medical devices or transmission through the hands of healthcare providers [8]. These pathogens include *coagulase*-negative *Staphylococcus*, *Staphylococcus aureus*, a wide range of gram-negative pathogens, including *Escherichia coli*, *Klebsiella* spp., *Pseudomonas* spp., and fungi [3,9]. Nevertheless, microbiology differs considerably among different geographic regions, with an increased prevalence of gram-negative pathogens reported in LMICs [10,11,12,13].

### 1.2. Antimicrobial Resistance in Neonatal Sepsis

Antimicrobial resistance is a global concern, with an increasing number of reports of multidrug-resistant (MDR) infections in pediatric and adult patients. An increase in the prevalence of colonization and infection with MDR pathogens has also been reported in the neonatal population, particularly in LMICs. The increased prevalence of MDR pathogens in LMICs is likely to be multifactorial in nature. The factors include inadequate sanitation and hygiene, the high burden of infectious diseases associated with limited access to healthcare, and the irrational and often inappropriate use of antibiotics [14,15]. It has been demonstrated that half of the pathogens causing severe infections in neonates are resistant to the first- and second-line recommended treatments, ampicillin, gentamicin, and third-generation cephalosporins [16]. A recent observational study investigating the burden of antimicrobial resistance in 39 NICUs from 12 LMICs reported up to 84% cephalosporin resistance and up to 80% carbapenem resistance among gram-negative pathogens causing neonatal sepsis [17]. It is estimated that 31% of neonatal sepsis mortality is due to MDR pathogens [18].

In previous decades, MDR gram-positive infections were considered the primary concern in NICUs; however, in recent years, the prevalence of MDR gram-negative pathogens has increased, and they are regarded as significant threats due to escalating resistance and a paucity of treatment options [14,19]. Extended-spectrum beta-lactamase-producing and carbapenem-resistant (CRE) *Enterobacterales* are increasingly reported to cause outbreaks in NICUs and are associated with significant morbidity and mortality [14,16,20]. In the USA in 2019, the Centers for Disease Control and Prevention (CDC) threat report included CRE-*Enterobacterales* and MDR-*Pseudomonas aeruginosa* as urgent threats and ESBL-*Enterobacterales* as serious threats [21]. These pathogens, transmitted either vertically or through contaminated equipment and indwelling devices, are implicated in hospital-acquired infections. The 2024 report indicated a rising prevalence of hospital-acquired infections caused by these pathogens [22]. A particular concern regarding neonatal MDR infections is the limited therapeutic options, as the majority of newer antibiotics targeting these infections are not authorized for neonatal use [23].

### 1.3. Drug Resistance in Gram-Negative Bacteria

#### 1.3.1. Antibiotic Resistance in Enterobacterales

The primary mechanism of antibiotic resistance in *Enterobacterales* is the production of beta-lactamase enzymes that render beta-lactam antibiotics ineffective by hydrolysis of the beta-lactam ring. Beta-lactamases are encoded by chromosomal genes or genes acquired on plasmids or transposons. Classification of beta-lactamases according to the Ambler system is based on their primary structure (Table 1) [24,25]. ESBL-producing genes are more frequently present in *Escherichia coli*, *Klebsiella pneumoniae*, *Klebsiella oxytoca*, and *Proteus mirabilis* [3].

The increasing prevalence of ESBL-producing *Enterobacterales* has resulted in a greater utilization of carbapenems. The primary mechanisms responsible for carbapenem resistance are the production of carbapenemases, which are carbapenem-hydrolyzing enzymes, and the combination of structural mutations and beta-lactamase activity, predominantly ESBLs encoded by plasmids and AmpC cephalosporinases [26]. ESBLs and AmpC have been demonstrated to result in carbapenem resistance when combined with porin mutations, which are membrane proteins found in gram-negative bacteria. Porin mutations have been shown to impede the diffusion of antibiotics across bacterial membranes, thereby enabling beta-lactamases to exert their hydrolyzing activity [3,16]. The most prevalent pathogen capable of producing carbapenem-resistant enzymes is *Klebsiella pneumoniae*. Ambler class A serine *Klebsiella pneumoniae* carbapenemase (KPC) is the most widespread carbapenemase.

#### 1.3.2. MDR *Pseudomonas aeruginosa*

*Pseudomonas aeruginosa* isolates can exhibit resistance to multiple antibiotics through a variety of intrinsic and acquired mechanisms. The intrinsic resistance mechanisms include diminished outer membrane permeability, efflux systems, and the production of enzymes with the capacity to inhibit antibiotics. Certain antibiotics, such as beta-lactams, penetrate the membrane through porin channels, and modification of these channels impedes this process. Furthermore, the accumulation of antibiotics can be mitigated by active export through membrane efflux channels [27,28,29]. Acquired resistance mechanisms include the horizontal transfer of genes capable of producing aminoglycoside-modifying enzymes or beta-lactamases and mutations leading to beta-lactamase production, overexpression of efflux pumps, or modification of porins [27]. *Pseudomonas aeruginosa* has been documented as a producer of various beta-lactamases, including Ambler classes A, B, C, and D. Among these, penicillinases of class A are the most prevalent [30].

### 1.4. Management of MDR Infections

The necessity to establish an efficacious treatment to combat this global threat has resulted in repurposing existing antibiotics, such as colistin and fosfomycin, and developing novel ones, predominantly beta-lactam/beta-lactamase inhibitors. Four novel beta-lactam/beta-lactamase inhibitors have been recently developed and approved for the treatment of MDR gram-negative infections: ceftazidime/avibactam, ceftolozane/tazobactam, meropenem/vaborbactam, and imipenem/cilastatin/relebactam [31,32].

However, data concerning the neonatal population is limited, and the therapeutic options are extremely restricted. Repurposed antibiotics, such as colistin, have been employed in the treatment of MDR gram-negative sepsis, particularly in LMICs. Nevertheless, the safety profile of these medications is not reassuring. The increasing need to combat MDR pathogens, combined with the lack of clinical and pharmacokinetic studies in neonates, often leads to the administration of off-label drugs for use in the neonatal population by extrapolating data from studies conducted in pediatric or adult populations. Nevertheless, the safety of this approach cannot be assured due to the known physiological differences in this unique population and the expected altered pharmacokinetics compared to older patients [33,34]. Among the novel therapies developed against MDR gram-negative pathogens, ceftazidime/avibactam and ceftolozane/tazobactam are the only ones that have recently been authorized for use in the neonatal population [35,36,37,38,39]. The objective of this narrative review is to provide a summary of the extant literature regarding the use of ceftazidime/avibactam and ceftolozane/tazobactam in the neonatal population and to encourage their utilization in combating MDR infections in neonates.

## 2. Materials and Methods

The PubMed and Google Scholar databases were searched for relevant studies up to January 2025 using the following keywords: neonatal sepsis, multidrug-resistant gram-negative bacteria, beta-lactams/beta-lactamase inhibitors, ceftazidime/avibactam, and ceftolozane/tazobactam. We also screened the reference lists of the retrieved articles to identify relevant articles that the initial search might not have covered. Ultimately, 94 articles were found, and 67 were included in this narrative review, specifically randomized controlled trials (RCTs), systematic reviews, narrative reviews, observational studies, and case reports. Moreover, the reference lists of the retrieved articles were reviewed to assess for the presence of relevant studies that may not have been detected in the initial search. Table 2 describes the methodology used in this narrative review to identify the relevant literature.

## 3. Ceftazidime/Avibactam

### 3.1. Mechanism of Action/Spectrum of Activity

Ceftazidime/avibactam (CAZ-AVI) represents a highly promising combination of ceftazidime, a third-generation cephalosporin with broad-spectrum activity and efficacy against *Pseudomonas aeruginosa*, with avibactam, a novel beta-lactamase inhibitor. Avibactam, a diazabicyclooctane non-beta lactam, does not itself exert antimicrobial action but protects ceftazidime from beta-lactamase degradation, thereby rendering it potent against otherwise resistant pathogens. Inhibition of beta-lactamases is achieved by a two-step process in which covalent binding results in deacylation. Binding to beta-lactamase molecules is reversible, so that following deacylation, avibactam is recycled and can then inhibit additional beta-lactamase molecules [40,41,42,43]. Avibactam efficiently inhibits class A, class C, and certain class D beta-lactamases but not class B (metallo-B-lactamases) [42,44].

The combination of CAZ-AVI is effective against a wide range of gram-negative bacilli. *Enterobacterales*, including ESBL and AmpC-producing strains and several carbapenemase D-producing bacteria such as OXA-24, OXA-40, OXA-48, and OXA-69, are generally susceptible to CAZ-AVI [42]. For carbapenem-resistant *Enterobacterales* with OXA-type carbapenemases, CAZ-AVI is considered the treatment of choice [32]. In a large surveillance study of 34,062 *Enterobacterial* isolates, the susceptibility rate to CAZ-AVI was reported to be 99.5% [45]. Among carbapenem-resistant isolates, susceptibility to CAZ-AVI has been shown to be as high as 73% [46]. CAZ-AVI is highly effective against *Pseudomonas aeruginosa*, and susceptibility has been reported to be up to 97% [42,47]. In the context of multidrug-resistant and extensively drug-resistant (XDR) isolates, the susceptibility rate has been demonstrated to be 82.1% and 75.8%, respectively [47]. CAZ-AVI has limited activity against gram-positive pathogens, gram-negative anaerobes, and strains that produce class B carbapenemase. In a large in vitro study, Castanheira et al. demonstrated that only 11 out of more than 20,000 *Enterobacterial* isolates presented a CAZ-AVI MIC > 8 μg/mL and that two of those 11 resistant strains produced metallo-B-lactamases [48]. Nevertheless, co-administration with aztreonam is effective against *Enterobacterales* producing metallo-B-lactamases [49,50].

Since 2015, CAZ-AVI has been approved by the Food and Drug Administration (FDA) for complicated urinary tract infections (UTIs) and for the treatment of intra-abdominal infections in combination with metronidazole. In subsequent years, approval was extended to include hospital-acquired and ventilator-associated bacterial pneumonia [51]. In 2019, CAZ-AVI was approved for the pediatric population, including infants older than three months. Recently, the European Medicines Agency (EMA) and the FDA approved the use of CAZ-AVI in neonates and infants younger than three months to treat complicated intra-abdominal infections, complicated UTIs, and hospital-acquired and ventilator-associated bacterial pneumonia [38,52]. The approval includes preterm infants; however, the FDA approval statement specifies that the indications apply to infants with a gestational age above 31 weeks [38]. According to the EMA, CAZ-AVI is authorized for infections of any localization caused by aerobic gram-negative bacteria, for which limited treatment options exist [52].

### 3.2. Pharmacokinetics and Dosing

Pharmacokinetic studies have demonstrated that the combination of these two agents does not alter the pharmacokinetics of either substance [53]. Moreover, the two agents exhibit linearity and similarity in their pharmacokinetic characteristics [41,42]. Both ceftazidime and avibactam are characterized by low plasma protein binding (10% and 8%, respectively), short half-lives (approximately 1.5–2 h), a similar volume of distribution, and are excreted unchanged in the urine [42,52]. Since both components are primarily excreted by the kidneys, dose adjustment is necessary for patients with impaired renal function [42]. The available data on CAZ-AVI pharmacokinetics in the pediatric population is very limited. To date, only a single trial has been conducted in neonates [54].

The formulation of CAZ-AVI is composed of a 4:1 ratio [51]. As both ceftazidime and avibactam demonstrate time-dependent activity, a prolonged infusion of two hours has been proposed and is applied to optimize the antimicrobial effect [42]. The recommended dosing for the adult population is 2000 mg/500 mg of CAZ-AVI every 8 h, irrespective of the site of infection [52].

There is limited data on the pharmacokinetic profile of CAZ-AVI in the pediatric population. Bradley et al. conducted a phase 1 study to assess the pharmacokinetic profile of a single dose of CAZ-AVI in a pediatric population aged three months to 18 years. The study cohort (*n* = 32) was divided into four age groups, and the administered dosage of CAZ-AVI was 2000 mg/500 mg for participants with a body weight of >40 kg and 50 mg/kg/12.5 mg/kg for children with a body weight of <40 kg. The pharmacokinetic characteristics observed were similar across all age groups and comparable to those previously reported in healthy adults [55]. Based on this study and two subsequent phase 2 RCTs in which similar doses of CAZ-AVI every 8 h were administered in pediatric populations, except infants aged three to six months who received a dose of 40 mg/kg/10 mg/kg, Franzese et al. assessed CAZ-AVI exposure in children over the age of three months using a population pharmacokinetic model [56,57,58]. According to the results of this study, mean exposure and probability of pharmacodynamic target attainment (PTA) were comparable to those in adults, suggesting that similar efficacy and safety can be expected in the pediatric population with the recommended dosing regimen [58].

The currently recommended dosing regimen for the pediatric population is 50 mg/kg/12.5 mg/kg every 8 h, with a maximum dosage of 2000 mg/500 mg every 8 h for children and adolescents aged six months to 18 years and 40 mg/kg/10 mg/kg for infants aged three to six months [38,52]. As both ceftazidime and avibactam are renally eliminated, dose adjustment is recommended in patients with renal impairment, with a reduction depending on renal function [51]. According to the EMA approval, pediatric patients older than three months and according to the FDA approval, pediatric patients older than two years with renal impairment should have the daily dose reduced by half in patients with a GFR of 31–50 mL/min/1.73 m^2^ and to 37.5% of the standard dose in patients with a GFR of less than 30 mL/min/1.73 m^2^. The EMA recommendation for dose adjustment in patients aged three months to two years does not apply to patients with a GFR < 16 mL/min/1.73 m^2^. The FDA approval does not include a dosing recommendation for patients younger than two years of age with renal impairment [38,52].

Following the approval of CAZ-AVI for the pediatric population by the FDA and EMA in 2019 and 2020, respectively, both agencies expanded the approval to include neonates and infants less than three months of age in 2024. This decision was based on the results of a recent non-randomized, multicenter phase 2a study evaluating the pharmacokinetics and safety of CAZ-AVI in term and preterm neonates and infants less than three months of age [38,52,59]. The study recruited 46 neonates and infants with suspected or proven gram-negative infections, aged two to 89 days, and gestational age 31 weeks to term. Although, according to the study design, neonates with a gestational age of >26 weeks would have been recruited, only neonates with a gestational age of >31 weeks were enrolled. The study cohort was divided into two groups: 25 infants received a single dose of CAZ-AVI, while 21 received multiple doses, with a median duration of treatment of seven days. The administered dose of CAZ-AVI was 30 mg/kg/7.5 mg/kg for term infants aged >28 days and preterm infants with a corrected age > 44 weeks, and 20 mg/kg/5 mg/kg for the younger term and preterm neonates. This dose was administered every 8 h in the group that received multiple doses. The authors concluded that plasma concentrations in both groups were comparable to those previously reported in pediatric cohorts and that the drug was safe and well-tolerated [59].

Current approvals, both EMA and FDA, recommend the aforementioned dosing regimen for neonates and young infants: 30 mg/kg/7.5 mg/kg every 8 h for term neonates > 28 days to three months and preterm neonates with postmenstrual age (the sum of gestational age and chronological age in weeks) > 44 weeks to 53 weeks and 20 mg/kg/5 mg/kg for younger neonates [38,52]. However, according to the FDA label, CAZ-AVI is approved for preterm neonates with a gestational age above 31 weeks [45]. Due to insufficient data, no dosing regimen has been recommended for neonates and young infants with renal impairment [38,52].

### 3.3. Efficacy

In the pediatric population older than three months, the efficacy of CAZ-AVI plus metronidazole and CAZ-AVI monotherapy in the treatment of complicated intra-abdominal infections and complicated UTIs, respectively, has been demonstrated in two randomized control trials [56,57]. However, CAZ-AVI use in the neonatal population has not been studied in RCTs or prospective clinical trials, and data on its efficacy in this unique population is limited to case reports and case series (Table 3).

Iosifidis et al. retrospectively described six cases of neonates, including five very preterm neonates, treated with CAZ-AVI for proven bloodstream infection with *CRE Klebsiella pneumoniae* (3/6) or sepsis and colonization with *CRE Klebsiella pneumoniae*. A favorable clinical and microbiological outcome was reported in all cases [60]. Ftergioti et al. recently reported on 21 neonates with a median gestational age of 29 weeks treated with 31 courses of CAZ-AVI. In this cohort, 61% of courses were administered empirically due to sepsis and known colonization with carbapenemase-producing *Klebsiella pneumoniae*. Twelve cases of bacteremia, two cases of UTI, and one case of ventilator-associated pneumonia were diagnosed in neonates receiving targeted treatment, and the most commonly isolated pathogen was *CRE Klebsiella pneumoniae* in 83% of cases. All neonates received multiple antibiotics prior to or concomitantly with CAZ-AVI. A favorable clinical response was observed in 73% of CAZ-AVI neonates. However, 24% of these critically ill neonates in this cohort died [61].

In another cohort of eight preterm neonates with bloodstream infections caused by ESBL *Klebsiella pneumoniae* and *CRE Klebsiella pneumoniae*, sterilization of blood cultures was reported after 48 h of treatment with CAZ-AVI 40 mg/kg/day/10 mg/kg/day tid combined with fosfomycin or amikacin [62]. Notably, although the seven ESBL *Klebsiella pneumoniae* isolates were susceptible to carbapenems, no clinical or microbiological response was observed following treatment with imipenem or meropenem, and CAZ-AVI was administered as salvage therapy. The authors hypothesized that this could be due to incomplete clearance of bacteremia and the development of antibiotic-resistant strains or heteroresistance [62].

Coskun et al. reported a case of a preterm neonate with UTI caused by pan-drug (PDR) resistant *Klebsiella pneumoniae* successfully treated with CAZ-AVI monotherapy for 10 days [63]. The efficacy of CAZ-AVI in the treatment of UTIs has been demonstrated in both pediatric and adult populations [57]. There is limited data about the effectiveness of CAZ-AVI in the treatment of central nervous system (CNS) infections. The penetration of CAZ-AVI into the cerebrospinal fluid was shown to be 38% in an experimental rabbit model [51]. Two cases of preterm neonates with multidrug-resistant *Klebsiella pneumoniae* meningitis successfully treated after 21 and 22 days of CAZ-AVI have been described [64,65]. It is noteworthy that in the case described by Afsour et al., the only drug co-administered with CAZ-AVI was colistin, which is known to have a limited ability to penetrate the cerebrospinal fluid, even in the presence of meningeal inflammation [64,66,67]. Indeed, there is increasing evidence supporting the efficacy of CAZ-AVI in the treatment of CNS infections caused by MDR gram-negative pathogens [68]. Pu et al. recently reported the successful treatment of *CRE Klebsiella pneumoniae* osteoarthritis in two preterm neonates with CAZ-AVI and surgical incision and drainage [69]. Growing evidence suggests that CAZ-AVI is an effective option for bone and joint infections caused by multidrug-resistant gram-negative bacteria [70,71].

**Table 3:** Published data on the use of ceftazidime/avibactam in neonates.

Author	Population	Pathogen	Site of Infection	Dosage	Duration	Concomitant Antibiotics	Efficacy	Potentially Treatment-Related Adverse Effects
Iosifidis, 2019 [60]	6 neonates (GA: 25 + 5–37 + 3, CA: 6–134)	XDR/PDR *Klebsiella Pneumoniae*	Suspected/proven bloodstream infection	50 mg/kg/12.5 mg/kg tid	4–21 days	At least three of the following antibiotics: carbapenems, fosfomycin, amikacin, colistin	Microbiological/clinical response of all neonates	Hypomagnesemia (2) Direct hyperbilirubinemia (1)
Esposito, 2019 [65]	1 preterm neonate (2 courses)	KPC *Klebsiella Pneumoniae*	CSF	75 mg/kg/20 mg/kg tid during the first 8 days and 25 mg/kg/6.5 mg/kg tid thereafter	25 days	Fosfomycin meropenem	Microbiological/clinical response	Mild thrombocytopenia (high-dose)
		KPC *Klebsiella Pneumoniae*	Bloodstream	25 mg/kg/6.5 mg/kg tid	22 days	Fosfomycin meropenem	Microbiological/clinical response	No
Coskun, 2020 [63]	1 neonate (CA: 25 days, GA: 27 weeks)	PDR *Klebsiella Pneumoniae*	UTI	40 mg/kg/10 mg/kg tid	10 days	Monotherapy	Microbiological/clinical response	Glucosuria
Asfour, 2022 [64]	1 neonate (CA: 17 days, GA: 27 weeks)	*CRE Klebsiella Pneumoniae*	CSF, bloodstream	50 mg/kg/12.5 mg/kg tid	21 days	Colistin	Microbiological/clinical response	No
	1 neonate (CA: 56 days, GA: 28 weeks)	*CRE Klebsiella Pneumoniae*	bloodstream	50 mg/kg/12.5 mg/kg tid	5 days	Amikacin	Microbiological cure/patient died (CA: 61 days)	Significant creatinine increase
Nascimento, 2022 [72]	1 neonate (CA: 46 days, GA: 29 weeks)	MDR *Klebsiella Pneumoniae*	bloodstream	40 mg/kg/10 mg/kg tid	14 days	Monotherapy	Microbiological/clinical response	Abdominal distention, hypokalemia
Pu, 2023 [69]	1 neonate (CA: 1 day, GA: 34 + 4 weeks)	*CRE Klebsiella Pneumoniae*	Bloodstream, osteoarthritis	40 mg/kg/10 mg/kg tid	14 days	Monotherapy	Microbiological/clinical response	No
	1 neonate (CA: 45 days, GA: 32 + 4 weeks)	*CRE Klebsiella Pneumoniae*	Puncture fluid (Hip arthritis, femoral osteomyelitis)	40 mg/kg/10 mg/kg tid	28 days	Monotherapy	Microbiological/clinical response	No
Marino, 2023 [62]	8 neonates (median GA 26.5 weeks)	7 ESBL *Klebsiella Pneumoniae* 1 *CRE Klebsiella Pneumoniae*	bloodstream	40 mg/kg/10 mg/kg tid	7–18 days	7 fosfomycin 1 amikacin	Microbiological/clinical response	No
Mangarov, 2023 [73]	1 neonate (CA: 24 days, GA: 36 weeks)	MDR *Klebsiella Pneumoniae*	bloodstream	40 mg/kg/10 mg/kg tid	17 days	Imipenem/cilastatin metronidazole	Microbiological/clinical response	No
Ftergioti, 2024 [61]	21 neonates (median GA: 29 + 2, median CA: 44 days), 31 courses	*CRE Klebsiella Pneumoniae*, XDR A. baumannii	12 bloodstream, 2 UTI, 1 VAP 61% empirical	20 mg/kg/5 mg/kg tid to 50 mg/kg/12.5 mg/kg tid	10 days (median)	In most cases, concomitant antibiotics were administered, including: Colistin Tigecycline Fosfomycin Amicakin	Clinical response >74% 5 deaths	No
Chen, 2024 [74]	1 neonate (CA: 25 days, GA: 32 weeks)	ESBL, OXA-48 *Klebsiella Pneumoniae*	bloodstream	50 mg/kg/12.5 mg/kg tid	15 days	Aztreonam fosfomycin	Microbiological/clinical response	No

CA: chronological age; GA: gestational age; XDR: extensively drug-resistant; MDR: multidrug-resistant; PDR: pandrug-resistant; KPC: *Klebsiella pneumoniae* carbapenemase; ESBL: Extended-spectrum beta-lactamase; CRE: carbapenem-resistant; CSF: cerebrospinal fluid; UTI: urinary tract infections; VAP: ventilator-associated pneumonia; tid: three times daily.

### 3.4. Safety

The most commonly reported adverse effects of CAZ-AVI in the pediatric population are gastrointestinal disturbances, including abdominal pain, diarrhea, and vomiting [56,57]. Based on the limited data available in neonates, no serious adverse events attributable to CAZ-AVI administration have been reported (Table 3).

The most commonly reported adverse reactions in patients younger than three months are increased transaminases and vomiting [38]. In a preterm neonate with a gestational age of 27 weeks, treated with CAZ-AVI due to PDR *Klebsiella pneumoniae* UTI, glucosuria was observed with normal blood glucose levels. In the absence of any evidence of a concomitant disease that might have resulted in glucosuria, and given that the neonate had not been administered any other medications at that time, the authors hypothesized that glucosuria could be attributable to reversible tubular dysfunction caused by CAZ-AVI. Notably, glucosuria progressively disappeared five days after cessation of treatment [63]. In another cohort, two out of six neonates treated with CAZ-AVI at a dose of 50 mg/kg/12.5 mg/kg every 8 h developed hypomagnesemia within 48 h of initiation of treatment with CAZ-AVI, fosfomycin, and colistin, which was treated with an increase in supplemental magnesium. In addition, one neonate in the same cohort developed direct hyperbilirubinemia, which resolved two weeks after sepsis. However, the authors state that no serious adverse effects requiring treatment discontinuation or dose modification were observed in this population, which, except for one neonate, consisted mainly of very preterm neonates (gestational age 25–32 weeks) [60]. A significant increase in serum creatinine was observed in a preterm infant treated with CAZ-AVI for *CRE Klebsiella pneumoniae* sepsis. However, a causal relationship with CAZ-AVI treatment cannot be assumed, as amikacin was concomitantly administered, and the neonate and the acute kidney injury could be a consequence of multi-organ dysfunction during the sepsis episode [64]. In the largest published cohort of 21 neonates treated with CAZ-AVI, Ftergioti et al. concluded that no adverse events could be attributed to CAZ-AVI use. This is due to the fact that all neonates were critically ill and concomitantly receiving other antibiotics with an uncertain safety profile [61].

### 3.5. Clinical Points

- CAZ-AVI is highly effective against ESBL, CRE *Enterobacterales*, and MDR/XDR *Pseudomonas aeruginosa.*
- Although CAZ-AVI is currently approved for the treatment of complicated intra-abdominal infections, UTIs, and nosocomial pneumonia, it can also be used for the treatment of aerobic gram-negative infections of any localization when treatment options are limited.
- The safety profile of CAZ-AVI has been demonstrated to be favorable in the neonatal population.

## 4. Ceftolozane/Tazobactam

### 4.1. Mechanism of Action/Spectrum of Activity

Ceftolozane/tazobactam (CEF-TAZ) is a novel combination of the fifth-generation semi-synthetic antipseudomonal cephalosporin ceftolozane with the well-established beta-lactamase inhibitor tazobactam [32]. Ceftolozane is distinguished by its high affinity for the penicillin-binding proteins of *Pseudomonas aeruginosa*, its enhanced stability to the class C *Pseudomonas*-derived cephalosporinase (AmpC), and its capacity to be less affected by alterations in the porin permeability or efflux pumps [31,75]. Tazobactam, a sulfone-based inhibitor, acts by irreversibly binding and inhibiting beta-lactamases. It is active against a limited spectrum of Ambler class A carbapenemases, excluding KPC, but not against class B metallo-B-lactamases or C oxacillinases [31,41,51]. CEF-TAZ is potent against common gram-negative pathogens, *Pseudomonas aeruginosa*, some ESBL *Enterobacterales*, some anaerobes including *Bacteroides fragilis*, and some *Streptococcus* spp. Conversely, its activity against ESBL-producing *Klebsiella pneumoniae*, *carbapenemase*-producing *Enterobacterales*, and anaerobic gram-positive cocci is limited [42].

CEF-TAZ is particularly active against *Pseudomonas aeruginosa*, including multidrug-resistant strains. An in vitro study demonstrated that CEF-TAZ exhibited a 91% inhibitory activity against carbapenem-resistant *Pseudomonas aeruginosa* strains. This activity was higher than the 81% activity observed with CAZ-AVI [76]. Humphries et al. analyzed 309 clinical isolates in a study that compared the activity of CEF-TAZ and CAZ-AVI against beta-lactam-resistant strains of *Pseudomonas aeruginosa*. Despite the high activity demonstrated by both agents, it was observed that 13% of the isolates exhibited susceptibility exclusively to CEF-TAZ, while 0.6% demonstrated susceptibility solely to CAZ-AVI [77]. A subsequent study investigated the susceptibility of 6836 *Pseudomonas aeruginosa* isolates to various antibiotics. CEF-TAZ was shown to be the second most potent antibiotic against *Pseudomonas aeruginosa*, with 93.5% of the isolates being susceptible, following colistin, in which 99.8% of isolates were susceptible. It is noteworthy that among the 16 colistin-resistant isolates, 81.2% were susceptible to CEF-TAZ [77]. Among XDR *Pseudomonas aeruginosa* strains, 82.9% susceptibility to CEF-TAZ has been reported [78]. Shortidge et al. investigated 6240 g-negative isolates from pediatric patients and reported a 94.6% susceptibility of *Enterobacterales* to CEF-TAZ and 97.4% of *Pseudomonas aeruginosa* isolates. CEF-TAZ was identified as the most potent cephalosporin against *Enterobacterales* and the most potent beta-lactam against *Pseudomonas aeruginosa* [79]. The lack of activity of CEF-TAZ against carbapenemase-producing isolates has been demonstrated [80,81].

CEF-TAZ has been approved by the FDA and EMA since 2014 and 2015, respectively. Initially, the approval included only adults with complicated UTIs or intra-abdominal infections in combination with metronidazole. Since 2019, the approval has been extended to adults with hospital-acquired and ventilator-associated pneumonia. In 2022, CEF-TAZ was approved for use in the pediatric population. The approval was granted for pediatric patients from birth with a GFR > 50 mL/min/1.73 m^2^ with complicated UTI or complicated intra-abdominal infection in combination with metronidazole. While the authorization extends to neonates, it is specified that this applies to neonates with a gestational age above 32 weeks and after the first postnatal week [39,51,82].

### 4.2. Pharmacokinetics and Dosing

Data from pharmacokinetic studies conducted in adult populations has demonstrated that the pharmacokinetic properties of ceftolozane and tazobactam remain unaltered when administered as a co-formulation. Both drugs are characterized by low protein binding, approximately 16–21% for ceftolozane and 30% for tazobactam. Ceftolozane and tazobactam are renally eliminated, and dose adjustment is required in patients with impaired renal function. More than 95% of ceftolozane is excreted unchanged by glomerular filtration. Tazobactam is excreted by glomerular filtration and tubular secretion, with 80% of the compound eliminated unchanged and 20% as the inactive metabolite M1. The mean half-life of ceftolozane in healthy adults is approximately three hours, whereas that of tazobactam is one hour. Ceftolozane and tazobactam are used as a 2:1 co-formulation [41,42,72,81].

Bradley et al. conducted a phase 1, non-comparative, open-label, multicenter study to assess the pharmacokinetics of a single dose of CEF-TAZ in pediatric patients with suspected or proven gram-negative infections [83]. The study cohort included patients from birth to 18 years of age and was divided into 6 age groups, and age-based doses were given. Neonates in the first postnatal week were not included in the study due to the well-documented poor renal function observed in this age group. Neonates and young infants aged less than three months were divided into two groups: the first group consisted of neonates born after 32 weeks of gestation, and the second group consisted of preterm neonates with a gestational age of less than 32 weeks. Following interim analysis, the doses of CEF-TAZ that were selected were 30 mg/kg/15 mg/kg for patients aged three months to 18 years and 20 mg/kg/10 mg/kg for term and preterm neonates, with the prerequisite that GFR was above 50 mL/min/1.73 m^2^. In preterm neonates with GFR < 50 mL/min/1.73 m^2^, a dose of CEF-TAZ of 12 mg/kg/6 mg/kg was selected. The pharmacokinetic parameters for both ceftolozane and tazobactam were comparable among pediatric patients. However, in the case of neonates and young infants, slightly altered pharmacokinetics were observed, which can be explained by age-related physiological changes. The higher extracellular water content in neonates, particularly preterm neonates, may result in the slightly increased volume of distribution observed compared to older children. In addition, the decrease in clearance and modest increase in terminal half-life observed in the youngest patients may be due to the immaturity of renal function in neonates and young infants [83,84].

Larson et al. conducted a population pharmacokinetic analysis using two-compartment linear models with first-order elimination to assess the exposure and pharmacokinetic target attainment of various dosing regimens in the pediatric population. They concluded that in patients younger than 12 years with a GFR > 50 mL/min/1.73 m^2^, including neonates and young infants, the optimal dose of CEF-TAZ is 20 mg/kg/10 mg/kg, administered by 1 h infusion every 8 h [85]. In a subsequent study, Roilides et al. evaluated the pharmacokinetic parameters of CEF-TAZ in a dose of 20 mg/kg/10 mg/kg every 8 h in 14 term neonates and young infants aged less than three months with pyelonephritis and reported similar ceftolozane and tazobactam exposures to those previously reported in adult and pediatric populations [86]. An ongoing multicenter, open-label, non-comparative Phase 1 clinical trial evaluates the pharmacokinetics and safety of CEF-TAZ in pediatric patients with nosocomial pneumonia (NCT04223752) [87].

According to the current EMA and FDA approvals, the recommended dosing regimen for neonates and children with complicated UTI or complicated intra-abdominal infection and a GFR > 50 mL/min/1.73 m^2^ is 20 mg/kg/10 mg/kg every 8 h. CEF-TAZ is not authorized for use in pediatric patients with renal impairment (GFR < 50 mL/min/1.73 m^2^), as dose adjustments in this population have not been established [39,82].

### 4.3. Efficacy

The efficacy of CEF-TAZ in adult populations with complicated UTIs, intra-abdominal infections, and nosocomial pneumonia has been demonstrated in several studies [88,89,90,91,92]. However, data regarding its efficacy in the pediatric population is limited, with the majority of available information being derived from two phase 2 RCTs (Table 4). Research in the neonatal population is particularly limited.

Roilides et al. conducted a multicenter phase 2 RCT to compare the efficacy of CEF-TAZ with meropenem in pediatric patients with complicated UTI. A total of 95 patients were enrolled, of whom 71 received CEF-TAZ, at a dose of 1 g/0.5 g every 8 h (patients > 12 years of age) or 20 mg/kg/10 mg/kg every 8 h (patients < 12 years of age), and 24 patients received meropenem. The most common diagnosis was pyelonephritis, with *Escherichia coli* being the most prevalent isolated pathogen (74.6% and 87.5% in CEF-TAZ and meropenem groups, respectively). Of the 74 isolated strains of *Escherichia coli*, 5.4% were identified as ESBL-producing. Both drugs were highly effective, with clinical cure reported in 94.4% of patients in the CEF-TAZ group and 100% in the meropenem group. Of note, two out of four cases reported as treatment failures in the CEF-TAZ group were classified as failures due to missing data. Following the cessation of treatment, the microbiological eradication rates were 93% and 95% in the CEF-TAZ and meropenem groups, respectively. All infections caused by ESBL-producing pathogens were successfully treated [93]. The authors conducted a subgroup analysis to assess the efficacy of CEF-TAZ in neonates and young infants aged less than three months. The subpopulation under analysis comprised 20 patients with pyelonephritis, 14 of whom were enrolled in the CEF-TAZ group and 6 in the meropenem group. *Escherichia coli* was the most common pathogen, isolated in 16 patients, followed by *Klebsiella pneumoniae* (2 patients), *Citrobacter* spp. (1 patient), and *Pseudomonas aeruginosa* (1 patient). Four patients, two in each group, presented with bacteremia. Clinical cure rates were 92.9% and 100% in the CEF-TAZ and meropenem groups, respectively. Four patients, two in each group, presented with bacteremia, and microbiologic eradication was achieved in all patients. The authors concluded that the efficacy of CEF-TAZ in neonates and young infants was comparable to that observed in the overall pediatric population [86].

Jackson et al. evaluated the efficacy of CEF-TAZ plus metronidazole in the treatment of complicated intra-abdominal infections in the pediatric population in a multicenter phase 2 RCT. A total of 91 patients were enrolled: 70 were treated with CEF-TAZ plus metronidazole, and 21 were treated with meropenem. The vast majority of patients (93.4%) were diagnosed with complicated appendicitis, and *Escherichia coli* was the most commonly isolated microorganism (65.9%), followed by *Pseudomonas aeruginosa* and *Bacteroides fragilis*; however, in more than half of the patients, polymicrobial infections were diagnosed. In the overall study population, clinical cure was reported in 80% of cases in the CEF-TAZ group and 100% in the meropenem group. As the authors note, the study was not powered for between-group comparisons, and differences between the two groups, including type of surgery and prevalence of polymicrobial infections, may have influenced clinical response rates. The reported efficacy of CEF-TAZ is comparable to that previously reported in adult populations with intra-abdominal infections [94].

Molloy et al. reported on 13 patients aged three months to 19 years with multidrug-resistant *Pseudomonas aeruginosa* treated with CEF-TAZ. In this group of patients, comprising children with severe comorbidities and heterogeneous infections—including seven patients with pneumonia, three patients with cystic fibrosis exacerbation, two with intra-abdominal infections, one with typhlitis and bowel perforation, and one with femoral osteomyelitis—clinical cure was achieved in all but one patient [95].

**Table 4:** Clinical studies on the use of ceftolozane/tazobactam in the pediatric population.

Author	Study Type	Population	Pathogens	Site of Infection	Dosage (Ceftolozane)	Duration	Efficacy	Potentially Treatment-Related Adverse Effects
Molloy, 2020 [95]	Case series	13 patients, 3 months–19 years	MDR *Pseudomonas aeruginosa*	Pneumonia (7) Cystic fibrosis exacerbation (3) Intra-abdominal infection (2) Osteomyelitis (1)	13.8–34.8 mg/kg tid	5–61 days	92.3% (12/13) clinical cure	Transaminitis (1) Neutropenia (1)
Roilides, 2023 [93]	Phase 2 RCT	71 patients, 7 days–18 years	*Escherichia coli* *Klebsiella pneumoniae* *Pseudomonas aeruginosa*	Complicated UTI	20 mg/kg tid (<12 years) 1 g tid (>12 years)	6.1 days (mean)	94.4% (67/71) clinical cure 93% (66/71) microbial eradication	Diarrhea (3) Increased appetite (3) Neutropenia (2) Increased aspartate aminotransferase (2)
Jackson, 2023 [94]	Phase 2 RCT	70 patients, 7 days–18 years	*Escherichia coli* *Pseudomonas aeruginosa* *Bacteroides fragilis*	Complicated intra-abdominal infection	20 mg/kg tid (<12 years) 1 g tid (>12 years) Plus metronidazole	6.4 days (mean)	80% clinical cure	Diarrhea (4) Increased aspartate aminotransferase (4) Increased alanine aminotransferase (3) Increased alkaline phosphatase (2) Dysgeusia (2)

RCT: Randomized controlled trial; MDR: multidrug-resistant; tid: three times daily; UTI: urinary tract infection

### 4.4. Safety

Evidence from research involving adult populations indicates that CEF-TAZ demonstrates a favorable safety profile with mild adverse effects, including gastrointestinal disturbances, elevated transaminases, and pyrexia [82]. The existing data indicates that CEF-TAZ demonstrates a comparable safety profile in pediatric patients to that observed in adults.

In a study by Bradley et al., 34 pediatric patients with suspected or proven gram-negative infections received a single dose of CEF-TAZ. Treatment-related adverse events were observed in two patients: one patient experienced dizziness during the infusion, and the other experienced tachycardia [84]. No treatment-related adverse events were observed in the 13 term and preterm neonates and infants less than three months of age enrolled in the study [84].

Roilides et al. compared the safety of CEF-TAZ with meropenem in pediatric patients with UTI in a phase 2 RCT, and both drugs were reported to have a comparable safety profile. The most frequently reported adverse effects in the CEF-TAZ group were gastrointestinal disturbances, neutropenia, and increased transaminases [87]. These findings are consistent with those of a subsequent RCT in a pediatric population with complicated intra-abdominal infections treated with CEF-TAZ combined with metronidazole [94]. In the subgroup of neonates and young infants (aged less than three months) who received CEF-TAZ at a dose of 20 mg/kg/10 mg/kg every 8 h, with a mean duration of treatment of 5.9 days, no severe side effects were reported. Three of the sixteen infants developed drug-induced neutropenia. Two were classified as moderate neutropenia and one as severe, lasting 6.6 weeks [86].

Data on the safety of CEF-TAZ in neonates is limited. However, based on the available literature, CEF-TAZ appears to have a favorable safety profile in the neonatal population.

### 4.5. Clinical Points

- CEF-TAZ is effective against various ESBL *Enterobacterales* and some anaerobes.
- This combination is very potent against MDR and XDR *Pseudomonas aeruginosa*.
- It is approved for complicated UTI or intra-abdominal infection combined with metronidazole. However, it has also been effective in infections of other sites caused by susceptible pathogens.
- There have been no reports of safety concerns in the neonatal population.
- CEF-TAZ and CAZ-AVI have both similarities and differences and represent valuable options for the treatment of MDR gram-negative infections in neonates (Table 5).

## 5. Limitations

Despite the fact that extensive literature research was conducted during the preparation of this review, given its narrative nature, a standardized literature search was not performed, which could potentially lead to selection bias.

The existing literature on the use of CAZ-AVI and CEF-TAZ in the neonatal population is relatively limited. The available data on CAZ-AVI is primarily retrieved from case reports and case series, and no RCTs or prospective trials have been conducted. Extrapolation of data from case reports and case series has several limitations, and the interpretation of findings should be carefully considered to avoid overstatement and generalization. Conversely, there are no published case reports on the use of CEF-TAZ in the neonatal population, and therefore, there is no data on the use of CEF-TAZ in neonates in daily clinical practice. The available data comes exclusively from two RCTs.

## 6. Conclusions

Antibiotic resistance represents a growing global threat. In order to combat this, there has been an increasing use of repurposed older antibiotics and the development of novel agents, such as beta-lactam/beta-lactamase inhibitors. However, the limited therapeutic options available for the treatment of MDR gram-negative pathogens in the neonatal population render the clinical management of such cases particularly challenging. Due to the paucity of data, it is common practice to prescribe off-label antibiotics for neonates using extrapolated data from studies in adult or pediatric populations, despite the fact that it is well established that pharmacokinetics in this particular population differ from those of older patients due to physiological changes.

Ceftazidime/avibactam and ceftolozane/tazobactam have been approved for use in neonates for the treatment of MDR and XDR gram-negative infections. It has been shown that both agents are highly effective against MDR/XDR *Pseudomonas aeruginosa*; however, CEF-TAZ has been demonstrated as the most potent agent. In the context of ESBL-producing bacteria, CAZ-AVI demonstrates a more extensive spectrum of activity. For carbapenemase-producing *Enterobacterales*, where CEF-TAZ is inactive, CAZ-AVI is the preferred treatment choice. However, future studies will provide additional data on these two potent agents’ pharmacokinetics, efficacy, and safety in term and preterm neonates.

In consideration of the escalating prevalence of antibiotic-resistant infections, there is an imperative for future studies to investigate the utility of other novel agents for the treatment of MDR pathogens that are already in use among older patients to facilitate the safe use of these agents in neonates.

## Figures and Tables

**Table 1:** Ambler classification of beta-lactamases and the corresponding beta-lactamase inhibitors that can inhibit them and be used in combination with antibiotics to extend their antimicrobial spectrum [24,25].

Ambler Class	Active Site	Type of β-Lactamase	Representative Enzyme	β-Lactamase Inhibitor
Class A	Serine	penicillinases	KPC, CTX-M	avibactam, relebactam, vaborbactam
Class B	Zinc	metallo-B-lactamases	NDM, VIM	None
Class C	serine	cephalosporinases	AmpC	avibactam, relebactam
Class D	Serine	oxacillinases	Oxa-48	avibactam

KPC: *Klebsiella pneumoniae* carbapenemase; NDM: Νew Delhi metallo-b-lactamases; VIM: Verona integron-encoded metallo-b-lactamases.

**Table 2:** The literature search strategy.

Database	Pubmed, Google Scholar
Timeframe	Up to January 2025
Keywords	neonatal sepsis, multidrug-resistant gram-negative bacteria, beta-lactams/beta-lactamase inhibitors, ceftazidime/avibactam, ceftolozane/tazobactam
Inclusion and exclusion criteria	Only full-text articles written in the English language were included

**Table 5:** Comparison of the characteristics of ceftazidime/avibactam and ceftolozane/tazobactam.

	Ceftazidime/Avibactam	Ceftolozane/Tazobactam
Classification of pharmaceutical substances.	Third-generation cephalosporin/diazabicyclooctane non-beta-lactam inhibitor	Fifth-generation cephalosporin/ sulfone-based inhibitor
Pharmacokinetic properties	Low plasma protein binding Short half-life Both compounds are renally eliminated	Low plasma protein binding Short half-life Both compounds are renally eliminated
Antimicrobial spectrum	Gram-negative *Enterobacterales* (including ESBL and AmpC) MDR/XDR *Pseudomonas aeruginosa*	Gram-negative pathogens (including a range of ESBL *Enterobacterales)* MDR/XDR *Pseudomonas aeruginosa* Certain anaerobes (including *Bacteroides fragilis*) Certain *Streptococcus* spp.
Non-susceptible pathogens	Gram-positive pathogens Gram-negative anaerobes Strains producing class B carbapenemases	ESBL-producing *Klebsiella pneumoniae* Carbapenemase-producing *Enterobacterales* Anaerobic gram-positive cocci Strains that produce class B carbapenemase or class C oxacillinases
Approved indications in pediatric patients	Complicated intra-abdominal infections Complicated UTIs Hospital-acquired/ventilator-associated bacterial pneumonia	Complicated UTI Complicated intra-abdominal infection in combination with metronidazole
Other indications	Difficult to treat infections of any localization caused by susceptible pathogens	Difficult to treat infections of any localization caused by susceptible pathogens
Safety concerns	Mild transient adverse effects have been reported in neonates	Mild transient adverse effects have been reported in neonates

XDR: extensively drug-resistant; MDR: multidrug-resistant; ESBL: extended-spectrum beta-lactamase; UTI: urinary tract infection.
